# A giant basilar artery perforator aneurysm

**DOI:** 10.1016/j.radcr.2021.12.034

**Published:** 2022-01-14

**Authors:** Unal Mutlu, Hans Kortman, Issam Boukrab

**Affiliations:** a Department of Radiology and Nuclear Medicine, Erasmus University Medical Center, PO Box 2040, Rotterdam, CA, 3000 The Netherlands; b Department of Radiology and Nuclear Medicine, Neurovascular Expertise Center, St. Elisabeth Hospital, Hilvarenbeekseweg 60, Tilburg, GC, 5022 The Netherlands

**Keywords:** Intracranial Aneurysm, Subarachnoid Hemorrhage, Stents

## Abstract

Basilar artery perforator aneurysms (BAPA's) are a rare entity. Their natural history and treatment are unclear. We describe the largest BAPA reported thus far in literature in a 64-year-old Caucasian woman. This patient did not present with subarachnoid hemorrhage, but with left hemiparesis due to pontine ischemia. The aneurysm was initially misdiagnosed as a tumoral mass in a referring center. Angiography confirmed the presence of a BAPA and a flow diverter was successfully placed. This case shows us that a BAPA can mimic a tumoral mass and can cause ischemia due to mass effect without having ruptured. Both conservative and flow diverter placement seems viable treatment options. Individual patient characteristics and preferences should be considered in decision-making for treatment.

## Background

Basilar artery perforator aneurysms (BAPA's) were first described in 1996 by Ghowala et al. and are extremely rare [1]. About 80 cases have been reported in literature thus far, and their diagnosis and management are challenging [2], [3], [4], [5]. Because of their small size, detection on computed tomography angiography (CTA), magnetic resonance angiography (MRA) imaging, and digital subtraction angiography (DSA) is difficult and often repeated DSA is needed to confirm the diagnosis. Typically, the aneurysm neck is not visible or very small on imaging. Their natural history is largely unknown, and thus a symptomatic patient with a BAPA causes a therapeutic dilemma. Aneurysm rebleed increases the risk of morbidity and mortality, but endovascular treatment strategies like flow diverter and stenting may accompany high complication risks [6]. This risk is due to the eloquent anatomic location of BAPA's which has the potential for perforator occlusion or increased hemorrhage risk due to dual antiplatelet therapy.

## Case presentation

**Fig. 1:**
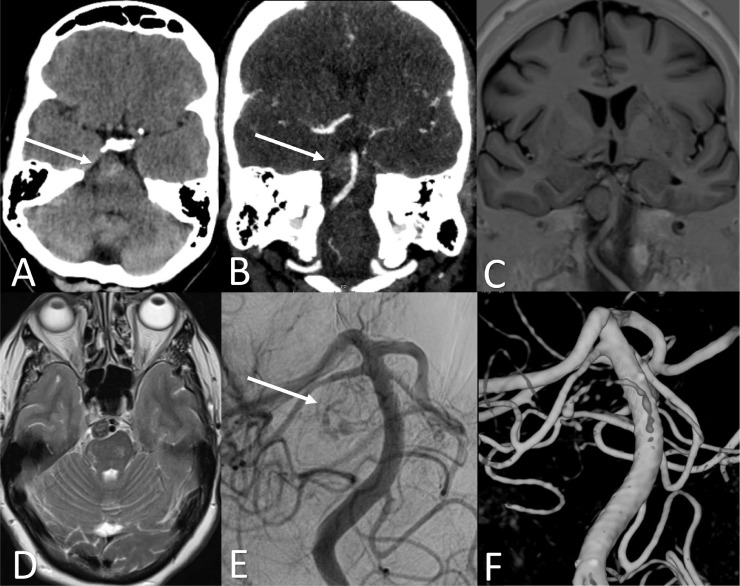
Different imaging modalities showing the basilar artery perforator aneurysm. (A) A prepontine mass on CT after 2 weeks of presentation; (B) CT with contrast did not show enhancement; (C) on T1 sequence MRI the aneurysm appeared iso-intens; (D) on T2 sequence MRI the aneurysm showed mixed intensities but mainly hypo-intens; (E) angiography showed a partially thrombosed aneurysm; (D) 3D reconstruction of the aneurysm on angiography.

A 64-year-old Caucasian woman with a history of diabetes mellitus type 2 was referred to our hospital with left hemiparesis, Hunt and Hess grade 4. CT-scan elsewhere demonstrated a mass at the cerebellopontine angle, which increased in density on CT imaging after two weeks (Fig. 1A), but did not show enhancement (Fig. 1B). Subsequent MRI on the same day demonstrated a lesion of 11 × 12 × 15 mm (length x width x height) with mass effect and MR-signal characteristics of thrombosis (Fig. 1C –D). We thought this lesion might be a thrombosed (pseudo-)aneurysm, and indeed, DSA performed on the same day corroborated our idea and demonstrated a partially thrombosed BAPA (Fig. 1E). Two days later a 4 × 23 mm flow diverter (MicroVention, Tustin, California) was deployed below the superior cerebellar arteries, covering the location of the perforator aneurysm. Control DSA demonstrated near total occlusion of the aneurysm with minimal filling of the aneurysm sac (Fig. 1F). Several hours after the procedure patient developed vision decrease. Subsequent CTA did not reveal a vessel occlusion, and no filling of the aneurysm sac. Likely a transient cortical blindness occurred due to injection of contrast. Dexamethasone bid 30 mg was described for 10 days. MRA scan after 2 days showed total occlusion of the aneurysm, without ischemia or new infarctions. Two days after she was completely healed from her blindness. Patient was discharged on day 9 of admission to a rehabilitation center, where she returned her original functional level with disability, Glasgow Outcome Scale 3. Follow-up MRA after 2 weeks of discharge showed total occlusion of the aneurysm and no complications.

## Discussion

We demonstrated one of the largest BAPA in a symptomatic patient without subarachnoid hemorrhage. Importantly, we showed a BAPA to be a diagnostic challenge due the clinical presentation, aneurysm size, and location, and partially thrombosed aneurysm. Hence, a BAPA could be a mimicker of a mass-like lesion.

From the literature search (See Supplemental), we found that BAPA's were not detected on initial imaging in about 60% of the cases, leading to a delay in diagnosis and treatment. Reasons may be spasm, intermittent thrombosis, or collapse of the aneurysm due to surrounding hemorrhage, but also unfamiliarity of clinicians with this entity. In about 15% of patients with spontaneous subarachnoid hemorrhage a cause is not identified on initial vascular imaging [7], yet it is not known to what extent BAPA's contribute to this percentage.

Interestingly, conservatively managed patients did not appear to have markedly higher percentages of ischemia or rebleed than treated patients. In fact, BAPA's seem to be more inclined causing an infarction while ‘berry aneurysm’ are known for their high risk of rebleed. Both patient groups treated conservatively or endovascularly demonstrated to have a 20% risk of developing ischemia. This may raise the question whether treatment is required at all. One may opt for a conservative treatment given the low probability of rebleed, but must be aware of ischemia. On the other side, endovascular stent placement may prevent rebleed while at the same time the anticoagulation therapy given postprocedural, prevents occlusion of the perforator arteries, and thereby maintaining an optimal balance.

Given the rarity of BAPA's, their natural course is unknown, and there is no consensus on any treatment strategy. Moreover, due to heterogeneity in how patients with BAPA's are investigated and treated, clinical equipoise exists. Future studies are required to clarify optimal treatment strategies.

## Conclusion

Basilar artery perforator aneurysms may be difficult to identify as a cause of subarachnoid hemorrhage. Our case demonstrates BAPA's may be a mimicker of a tumoral process without having ruptured. Hence, clinicians should consider an aneurysm in a prepontine mass. Individual patient characteristics and preferences should be considered in decision making for treatment.

## Patient consent

A written informed consent was obtained from the patient.

## Declaration of Competing Interest

The manuscript has not been submitted elsewhere nor published elsewhere in whole or in part. None of the authors has potential conflicts of interest related to this manuscript.

## Appendix Supplementary materials

Image, application 1
