# Supplementary material for: A giant basilar artery perforator aneurysm
Source: Radiol Case Rep. 2022 Jan 14;17(3):911–3. doi: 10.1016/j.radcr.2021.12.034 (PMC8762371; doi:10.1016/j.radcr.2021.12.034)
Supplement: Supplementary file 1 [file mmc1.docx]

| Supplemental table | | | | | | | | | | | |
| --- | --- | --- | --- | --- | --- | --- | --- | --- | --- | --- | --- |
| Case number | First author | Ischemia | Rebleed | Date | Age | Sex | Size, mm | Follow-up, months | Delayed Detection | Treatment | Ruptured |
|  | Ghogawala | No | No | 1996 | 56,00 | Female | 3,00 | 6,00 | Yes | Surgery | Yes |
|  | Hamel | No | No | 2005 | 44,00 | Male | - | 7,00 | No | Surgery | Yes |
|  | Sanchez | No | No | 2007 | 27,00 | Male | 6,00 | - | Yes | Surgery | Yes |
|  | Mathieson | No | No | 2010 | 51,00 | Male | 6,00 | - | No | Surgery | Yes |
|  | Gross | No | No | 2013 | 52,00 | Male | 4,00 | 12,00 | No | Surgery | Yes |
|  | Apok | Yes | No | 2013 | 64,00 | Male | 6,00 | 20,00 | Yes | Surgery | Yes |
|  | Sivakanthan | No | No | 2015 | 45,00 | Female | 2,00 | 6,00 | Yes | Surgery | Yes |
|  | Deshaies | No | No | 2011 | 47,00 | Female | 3,00 | 6,00 | Yes | Stent-in-stent | Yes |
|  | Chen | No | No | 2012 | 66,00 | Male | 7,00 | 24,00 | No | Coils | Yes |
|  | Chen | No | No | 2012 | 28,00 | Female | 10,00 | 18,00 | No | Coils | Yes |
|  | Nyberg | No | No | 2013 | 50,00 | Male | - | 14,00 | No | Stent-in-stent | Yes |
|  | Nyberg | No | No | 2013 | 70,00 | Female | 3,00 | 4,00 | Yes | Stent-in-stent | Yes |
|  | Ding | Yes | No | 2013 | 58,00 | Male | 2,00 | - | - | Conservative/failed | Yes |
|  | Chalouhi | No | No | 2014 | - | Female | 2,00 | 6,00 | Yes | Flow diverter | Yes |
|  | Peschillo | Yes | No | 2016 | - | - | 2,00 | 6,00 | No | Flow diverter | Yes |
|  | Peschillo | No | No | 2016 | - | - | 1,00 | 36,00 | Yes | Flow diverter + stent | Yes |
|  | Peschillo | No | No | 2016 | - | - | 2,00 | 6,00 | Yes | Flow diverter | Yes |
|  | Forbrig | Yes | Yes | 2016 | 59,00 | Male | 3,00 | 23,00 | Yes | Coils | Yes |
|  | Forbrig | Yes | No | 2016 | 71,00 | Male | 4,00 | 60,00 | No | Onyx | Yes |
|  | Forbrig | No | No | 2016 | 53,00 | Male | 1,00 | 6,00 | Yes | Conservative | Yes |
|  | Forbrig | No | No | 2016 | 72,00 | Male | 2,00 | 5,00 | Yes | Conservative | Yes |
|  | Forbrig | Yes | Yes | 2016 | 82,00 | Male | 2,00 | 6,00 | No | Conservative | Yes |
|  | Forbrig | Yes | No | 2016 | 71,00 | Female | 7,00 | 11,00 | No | Conservative/failed | Yes |
|  | Forbrig | Yes | No | 2016 | 65,00 | Male | 1,00 | 15,00 | Yes | Conservative | Yes |
|  | Forbrig | Yes | No | 2016 | 60,00 | Female | 3,00 | 78,00 | No | Conservative/failed | Yes |
|  | Finitsis | No | No | 2017 | 62,00 | Female | 1,00 | - | Yes | Flow diverter | Yes |
|  | Finitsis | No | No | 2017 | 59,00 | Male | 1,00 | - | Yes | Conservative | Yes |
|  | Finitsis | Yes | No | 2017 | 78,00 | Male | 3,00 | 12,00 | No | Conservative | Yes |
|  | Finitsis | No | No | 2017 | 53,00 | Female | 1,00 | 2,00 | Yes | Conservative | Yes |
|  | Buell | - | - | 2017 | - | - | 2,00 | 73,00 | - | Conservative/failed | Yes |
|  | Buell | - | - | 2017 | - | - | 1,00 | 2,00 | Yes | Conservative/failed | Yes |
|  | Buell | - | - | 2017 | - | - | 2,00 | 42,00 | Yes | Conservative | Yes |
|  | Buell | - | - | 2017 | - | - | 2,00 | 11,00 | Yes | Stent-in-stent | Yes |
|  | Buell | - | - | 2017 | - | - | 3,00 | 12,00 | Yes | Stent-in-stent | Yes |
|  | Buell | - | - | 2017 | - | - | 2,00 | 62,00 | Yes | Conservative | Yes |
|  | Buell | - | - | 2017 | - | - | 2,00 | - | - | Conservative | Yes |
|  | Satti | No | No | 2017 | 52,00 | Male | 4,00 | 7,00 | Yes | Stent-in-stent | Yes |
|  | Chau | No | No | 2017 | 53,00 | - | 2,00 | 6,00 | No | Stent-in-stent + coils | Yes |
|  | Chau | No | No | 2017 | 59,00 | - | 2,00 | 6,00 | Yes | Stent-in-stent | Yes |
|  | Chau | No | No | 2017 | 69,00 | Male | 3,00 | 12,00 | Yes | Conservative | Yes |
|  | Park | No | No | 2009 | 54,00 | Female | - | 16,00 | No | Conservative | Yes |
|  | Park | No | No | 2009 | 67,00 | Male | - | 16,00 | No | Conservative | Yes |
|  | Park | No | No | 2009 | 53,00 | Male | - | 1,00 | No | Conservative | Yes |
|  | Chavent | No | No | 2014 | 55,00 | Male | 2,00 | 6,00 | Yes | Conservative | Yes |
|  | Chavent | No | No | 2014 | 39,00 | Female | 3,00 | 12,00 | Yes | Conservative | Yes |
|  | Chavent | No | No | 2014 | 59,00 | Male | 1,00 | 6,00 | Yes | Conservative | Yes |
|  | Aboukais | No | No | 2016 | 67,00 | Male | 3,00 | 6,00 | Yes | Conservative | Yes |
|  | Daruwalla | No | No | 2016 | 76,00 | Male | 3,00 | - | No | Conservative | Yes |
|  | Bhogal | No | No | 2019 | 55,00 | Male | 2,00 | 9,00 | No | Flow diverter | Yes |
|  | Bhogal | No | No | 2019 | 65,00 | Male | 2,00 | 3,00 | Yes | Flow diverter | Yes |
|  | Bhogal | No | No | 2019 | 66,00 | Male | 2,00 | 12,00 | No | Flow diverter | Yes |
|  | Bhogal | Yes | No | 2019 | 41,00 | Female | 3,00 | 5,00 | No | Flow diverter | Yes |
|  | Bhogal | No | No | 2019 | 52,00 | Male | 2,00 | 4,00 | No | Flow diverter | Yes |
|  | Bhogal | No | No | 2019 | 39,00 | Female | 3,00 | 3,00 | No | Flow diverter | Yes |
|  | Bhogal | No | No | 2019 | 59,00 | Male | 2,00 | 3,00 | No | Conservative | Yes |
|  | Bhogal | - | - | 2019 | 57,00 | Male | 2,00 | - | Yes | Conservative | Yes |
|  | Bhogal | No | Yes | 2019 | 62,00 | Male | 1,00 | - | Yes | Conservative | Yes |
|  | Sahu | No | No | 2017 | 75,00 | Male | 2,00 | 3,00 | Yes | Double stenting | Yes |
|  | Sahu | No | No | 2017 | 58,00 | Male | 2,00 | 4,00 | Yes | Stenting | Yes |
|  | Sahu | No | No | 2017 | 16,00 | Male | 2,00 | - | Yes | Conservative | Yes |
|  | Sahu | - | - | 2017 | 32,00 | Male | 1,00 | - | Yes | Conservative | Yes |
|  | Sahu | - | - | 2017 | 65,00 | Female | 1,00 | - | No | Conservative | Yes |
|  | Sahu | - | - | 2017 | 35,00 | Female | 1,00 | - | Yes | Conservative | Yes |
|  | Lockwood | No | No | 2018 | 75,00 | Female | 3,00 | 5,00 | No | Coils | Yes |
|  | Sekar | Yes | No | 2019 | - | Female | 5,00 | 6,00 | Yes | Coils | Yes |
|  | Enomot | No | No | 2020 | 60,00 | Male | 3,00 | 2,00 | Yes | Conservative | Yes |
|  | Sattur | No | No | 2020 | 62,00 | Male | - | 3,00 | Yes | Stent-in-stent | Yes |
|  | Murata | No | No | 2020 | 34,00 | Female | - | 12,00 | No | Conservative | No |
|  | Zhang | No | No | 2019 | 37,00 | Female | - | 5,00 | - | Stent | Yes |
|  | Inoe | Yes | No | 2020 | 72,00 | Male | 2,00 | 2,00 | - | Stent | Yes |
|  | Grannan | Yes | No | 2014 | 65,00 | Female | - | 36,00 | Yes | Surgery | Yes |
|  | Grannan | - | - | 2014 | 60,00 | Male | 2,00 | 5,00 | Yes | Surgery | Yes |
|  | Shlobin | No | No | 2020 | 62,00 | Male | 1,00 | 30,00 | Yes | Conservative | Yes |
|  | Shlobin | No | No | 2020 | 48,00 | Female | 1,00 | 12,00 | Yes | Conservative | Yes |
|  | Ma | - | - | 2021 | - | - | - |  | - | Coiling/failed electr | Yes |
|  | Ma | - | - | 2021 | - | - | - | - | - | Electrothrombosis | Yes |
|  | Ma | - | - | 2021 | - | - | - | - | - | Electrothrombosis | Yes |
|  | Cox | No | No | 2021 | 53 | Male | 2 | 6 | Yes | Stent | Yes |
